# Early mobilisation in Windhoek intensive care units: Practices, attitudes and barriers

**DOI:** 10.4102/sajp.v81i1.2118

**Published:** 2025-01-31

**Authors:** Ilse du Plessis, Savarna Francis, Brenda Morrow

**Affiliations:** 1 Department of Health and Rehabilitation Sciences, Faculty of Health Sciences, University of Cape Town, Cape Town, South Africa; 2 Department of Paediatrics and Child Health, Faculty of Health Sciences, University of Cape Town, Cape Town, South Africa

**Keywords:** early mobilisation, barriers, facilitators, perceptions, knowledge, critical illness

## Abstract

**Background:**

Early mobilisation (EM) of critically ill patients in intensive care units (ICUs) has gained significant attention because of its potential to improve patient outcomes. Despite the recognised benefits of EM, implementation remains inconsistent.

**Objectives:**

To describe the knowledge, attitudes and practices of healthcare professionals regarding EM of critically ill patients in Windhoek.

**Method:**

A descriptive, cross-sectional design using a self-administered survey was conducted in Windhoek, Namibia, targeting nurses, doctors and physiotherapists working in private ICUs.

**Results:**

A total of 174 surveys were distributed, with a response rate of 24.1% (*n* = 42). Respondents included 21 nurses, 5 doctors and 13 physiotherapists. Most participants underestimated the incidence of ICU-acquired weakness and reported unfamiliarity with EM literature (*n* = 19, 51.4%). Furthermore, 25 respondents (67.6%) reported that patients were not automatically assessed for mobilisation, the majority reported requiring a doctor’s referral (*n* = 31, 83.8%). Mobility practices were conservative, especially when patients were intubated or in the presence of radial and femoral catheters. Major patient-level barriers included medical instability (*n* = 24, 72.7%) and excessive sedation (*n* = 18, 54.5%); whereas institutional barriers were the requirement for a doctor’s referral (*n* = 22, 64.7%) and no written guidelines or protocols for mobilisation (*n* = 16, 47.1%). Provider level barriers were that mobility is not perceived as important by some individuals (*n* = 18, 78.3%).

**Conclusion:**

Our study revealed knowledge gaps, conservative mobility practices and numerous barriers to EM implementation at the patient, provider and institutional levels.

**Clinical implications:**

The findings highlight the need for targeted education, training programmes, standardised mobility protocols and the establishment of a dedicated mobility champion to promote EM in Windhoek ICUs.

## Introduction

Early mobilisation (EM) of critically ill patients in intensive care units (ICUs) has gained significant attention over the past decade because of its potential to improve patient outcomes (Monsees et al. 2023; Zhang et al. 2019; Tipping et al. 2017). Prolonged immobility in the ICU is associated with numerous complications, including ICU-acquired weakness (ICUAW), which affects approximately 40% of patients undergoing mechanical ventilation for more than 48 h (Vanhorebeek, Latronico & Van den Berghe 2020; Yang et al. 2022). Intensive care unit-acquired weakness is linked to increased mortality, prolonged hospitalisation and impaired functional recovery (Meyer-Frießem et al. 2021). Recent studies have shown that EM may reduce the incidence of ICUAW, improve functional capacity and shorten the duration of ICU and hospital stay (Alaparthi et al. 2020; Monsees et al. 2023; Tipping et al. 2017). However, the optimal timing, intensity and patient selection criteria for EM are still under investigation (Menges et al., 2021).

Early mobilisation in ICUs is a multidisciplinary practice that involves collaboration among various healthcare professionals for the successful integration of EM in clinical practice (Dubb et al. 2016; Lang et al. 2020). Each group plays a crucial role in patient care, decision-making processes and communication related to mobilisation activities. Despite knowledge on the recognised benefits of EM, its implementation remains inconsistent across ICUs globally, with studies reporting low levels of mobilisation achieved, particularly for mechanically ventilated patients (Jolley et al. 2017; Tadyanemhandu, Van Aswegen & Ntsiea 2018; Tadyanemhandu, Van Aswegen & Ntsiea 2021b). The presence of medical devices and risk of dislodgement is often perceived as a barrier to EM, notwithstanding evidence supporting its safety when appropriate precautions are taken (Nydahl et al. 2017), highlighting a potential gap between current research and healthcare professionals’ knowledge in EM practices (Akhtar & Deshmukh 2021; Dubb et al. 2016; Tadyanemhandu et al. 2021b). Furthermore, EM is often hindered by barriers such as excessive sedation, the lack of protocols and limited staff resources (Bennion et al. 2024; Capell, Tipping & Hodgson 2019; Lin et al. 2020; Tadyanemhandu et al. 2018, 2021b). A broad range of barriers have been reported that encompasses patient and process-related factors, structural barriers as well as ICU culture (Costa et al. 2017; Dubb et al. 2016; Popoola et al. 2022; Tadyanemhandu, Van Aswegen & Ntsiea 2021a).

Unit specific contextual factors such as mobilisation protocols, staff to patient ratio, equipment, multidisciplinary wards rounds and the presence of a mobility champion, have all been found to be facilitators of EM (Albarrati et al. 2024a; Bennion et al. 2024; Lang et al. 2020). These factors can differ substantially from one unit to another, particularly in healthcare systems facing resource constraints and regional disparities, as is the case in Namibia.

Namibia, an upper middle-income country in southwestern Africa with a population of approximately 3 million (WHO 2018), faces unique challenges in its healthcare system, especially in intensive care. The country operates a dual healthcare system, with a public sector serving about 82% of the population and a private sector catering to the remaining 18% (Christians 2020). Intensive care units are primarily concentrated in the capital city, Windhoek, with limited availability in other regions.

The public sector hospital in Windhoek has a ICU with eight beds, while two participating private hospitals have eight and nine ICU beds, respectively (Tobi & Ogunbiyi 2024). The Namibian healthcare system faces significant challenges, including a shortage of healthcare professionals, particularly in specialised fields such as critical care (Nakweenda, Anthonie & Van der Heever 2022; WHO 2018). This shortage of healthcare professionals, with only 0.4 physicians and 2.9 nurses per 1000 population (WHO 2018), likely impacts the implementation of EM practices differently compared to other African countries or high-income countries. While there is a paucity of literature on EM in Africa, studies from neighbouring South Africa and Zimbabwe have reported resource-related barriers to EM, such as the lack of equipment and staffing (Tadyanemhandu et al. 2021a). However, Namibia’s unique healthcare landscape may present distinct challenges that have not been previously explored.

For our study, EM was defined as physiotherapist-directed rehabilitation started within 48 h of admission to the ICU (Koo et al. 2016). While studies have been conducted in various settings, our study aimed to explore the knowledge, attitudes and practices of nurses, doctors and physiotherapists regarding EM of critically ill patients in Windhoek ICUs, which has not been previously explored. The unique healthcare landscape, resource constraints and cultural factors in Namibia may influence EM practices differently compared to other countries. The primary objective was to assess the current state of EM practices and identify barriers to its implementation. Secondary objectives included evaluating the familiarity of healthcare professionals with EM guidelines and literature, and understanding their perceptions of the benefits and challenges associated with EM. Our study aims to identify these context-specific factors to provide insights that could inform targeted interventions and quality improvement programmes to enhance EM practices in ICUs.

## Research methods and design

### Study design

Our study employed a descriptive, cross-sectional, self-administered survey to assess the knowledge, attitudes and practices of healthcare professionals regarding EM in Windhoek ICUs.

The initial aim was to utilise total population sampling, a type of purposive sampling that involves examining the entire population of interest. Institutional approval requests were sent to both public and private facilities with ICUs with permission received only from two Windhoek facilities. Participation in the survey was voluntary, and written informed consent was obtained from all participants. Data were anonymised to ensure confidentiality.

The survey was conducted in Windhoek, targeting nurses, doctors and physiotherapists working in ICUs at Roman Catholic Hospital (9 bed ICU) and Mediclinic Windhoek (8 bed ICU) during August 2018. The Namibian Society of Physiotherapy also distributed the survey electronically to its members. Qualified nurses, doctors and physiotherapists with at least 1 year of post-qualification ICU working experience were included in our study and interns in ICU were excluded, this was to ensure sufficient exposure to ICU practices’ familiarity with the local context.

### Sample size calculation

The sample size was determined based on a guide for the design and conduct of self-administered surveys of clinicians created by Burns et al. (2008). Of the estimated 686 nurses, doctors and physiotherapists working in Namibian ICUs, 174 work at our study hospitals. Allowing a 10% margin of error, the minimum sample required was calculated to be 63 (https://www.surveymonkey.com/mp/sample-size-calculator/). The survey was sent to all eligible healthcare providers (*n* = 174) working in our study site ICUs, in order to obtain the highest possible return rate.

### Measurement instrument

The survey was designed to capture comprehensive data on the perceptions and practices related to EM. The survey was adapted from a validated tool developed by Koo et al. (2016) and included questions on clinician demographics, professional background, knowledge of EM, mobility practices, attitudes and perceptions, and perceived barriers to EM (Online Appendix 1). The survey was pilot tested for usability at an academic hospital in Cape Town, South Africa, where the survey was conducted with a group of 13 healthcare professionals consisting of five doctors, six ICU nurses and two physiotherapists. Adjustments were made based on feedback from pilot participants.

The primary outcome of the survey was to assess the current state of EM practices and identify barriers to their implementation. Secondary outcomes included evaluating the familiarity of healthcare professionals with EM guidelines and literature, and understanding their perceptions of the benefits and challenges associated with EM.

### Data collection and analysis

Data were collected for 6 months, through hard copies, distributed by the primary researcher, and an online survey platform. Intensive care unit managers assisted in distributing surveys to all staff on both day and night shifts, placing them in a collection box at the ICU administration station. Unit managers received weekly reminders about survey completion. In addition, a representative from the Namibian Society of Physiotherapy (NSP) distributed surveys to its members during a fun run event hosted by NSP and also emailed the survey to its members. Analyses were performed using *Statistica version* 13.2 (TIBCO Software Inc. (2016), Palo Alto, California, United States). statistical software. Descriptive statistics were used to summarise the profile of participants and their responses using frequencies and percentages. Continuous data were tested for normality using the Shapiro–Wilk test, and central tendency was presented according to distribution – means and standard deviations (s.d.) for normally distributed data, and medians (interquartile range, [IQR]) for non-parametric data. Incomplete survey responses were included in the analysis, indicated as ‘unanswered’ in the results section. Missing data were handled by using available case analysis.

### Ethical considerations

An application for full ethical approval was made to the Human Research Ethics Committee of the University of Cape Town and the consent was received on 28 February 2018. The ethics approval number is (HREC REF no 116/2018). Institutional approval was obtained from the Mediclinic Human Research Ethics Committee (Namibia) and participating hospitals.

## Results

### Participants’ characteristics

In all, 174 surveys were distributed to all doctors, nurses and physiotherapists working in our study hospital ICUs, with a response rate of 24.1% (*n* = 42), resulting in an adjusted margin of error (13.0%).

**FIGURE 1:**
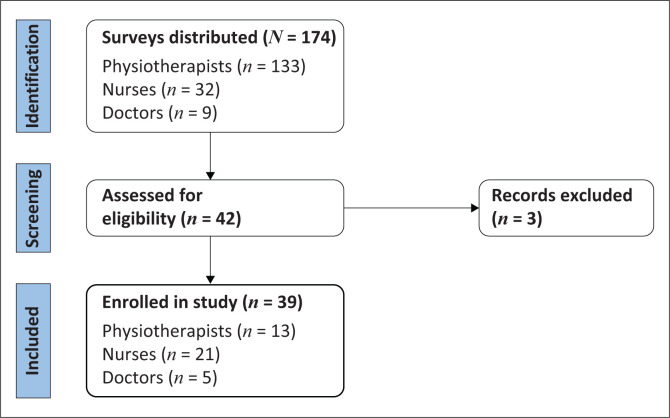
Enrolment process.

The respondents included 21 nurses (53.8%), 5 doctors (12.8%) and 13 physiotherapists (33.3%) (Figure 1). Most participants (*n* = 14, 42.4%) had between 1 year and 5 years of ICU experience, with a median of 7 years (IQR: 4–9). The majority of nurses (*n* = 17, 81%) received their undergraduate training at the University of Namibia, while physiotherapists (*n* = 9, 69.2%) and doctors (*n* = 4, 80%) were trained at various South African universities (Table 1).

**TABLE 1:** Participants’ demographical information.

Characteristic	Total number of responses		Nurses		Doctors		Physiotherapists	
	*n*	%	*n*	%	*n*	%	*n*	%
**Healthcare professional**	39	100.0	21	53.8	5	12.8	13	33.3
**Primary area of practice**								
Adult	33	84.6	20	95.2	4	80.0	9	69.2
Adult and paediatric	6	15.4	1	4.8	1	20.0	4	30.8
**Additional ICU training**								
Specialisation in ICU	8	20.5	8	38.0	0	0.0	0	0.0
Postgraduate ICU training	12	30.8	7	33.3	4	80.0	1	7.7
**Undergraduate training institution**								
University of Zimbabwe	2	5.1	0	0.0	0	0.0	1	7.7
University of the Western Cape	3	7.7	0	0.0	0	0.0	3	23.1
Stellenbosch University	5	12.8	0	0.0	2	40.0	3	23.1
University of Pretoria	4	10.3	0	0.0	1	20.0	3	23.1
University of the Free State	2	5.1	0	0.0	0	0.0	2	15.4
Dr MGR Medical University (India)	1	2.6	1	4.8	0	0.0	0	0.0
University of Cape Town	2	5.1	0	0.0	2	40.0	0	0.0
University of Namibia	17	43.6	17	81.0	0	0.0	0	0.0
Gordonia College (Upington)	1	2.6	1	4.8	0	0.0	0	0.0
United Bulawayo Hospitals Nursing College (Zimbabwe)	1	2.6	1	4.8	0	0.0	0	0.0
Mediclinic Tshwane Learning Centre (Pretoria)	1	2.6	1	4.8	0	0.0	0	0.0
**Years of experience**								
1–5	14	35.9	8	38.1	0	0.0	6	46.2
6–10	6	15.4	5	23.8	0	0.0	1	7.7
11–15	4	10.3	1	4.8	1	20.0	2	15.4
16–20	3	7.7	2	9.5	0	0.0	1	7.7
21–25	2	5.1	1	4.8	1	20.0	0	0.0
26–30	3	7.7	2	9.5	1	20.0	0	0.0
31–35	1	2.6	0	0.0	1	20.0	0	0.0
Unanswered	6	15.4	2	9.5	1	20.0	3	23.1

ICU, intensive care unit.

### Knowledge of early mobilisation

Of the 37 participants who answered the question on familiarity with published clinical trials and literature on EM, 18 (48.6%) reported being familiar, while 19 (51.4%) were not. Most participants underestimated the incidence of ICUAW, with only 6 (15.8%) selecting the correct answer (incidence > 40%). The majority of the physiotherapists (61.5%) and almost half of the nurses (45.0%) felt somewhat trained to mobilise mechanically ventilated patients. While 27 participants (69.2%) agreed with the statement that EM can improve patients’ functional independence, 25 (64.1%) agreed that EM can reduce the incidence of deep vein thrombosis and 18 (46.2%) participants agreed that EM is associated with reduced mortality at hospital discharge.

### Mobility practices

Most participants (*n* = 25, 64.1%) reported that patients were not automatically assessed for mobilisation and required a doctor’s referral (*n* = 31, 83.8%). Physicians/doctors were most often reported as the first practitioners to identify patients’ readiness for mobilisation (*n* = 24, 64.9%). Approximately half indicated that their ICUs did not have a mobility protocol (*n* = 19, 48.7%) or a mobility champion (*n* = 20, 51.3%). Sedation protocols were not routinely used, with many (*n* = 12, 31.6%) reporting they were used only ‘sometimes’.

The most commonly used techniques were manual chest physiotherapy (*n* = 9, 69.2%), bed mobility (*n* = 8, 61.5%) and pre-gait activities (*n* = 7, 53.8%).

### Attitudes and perceptions

The majority of participants (*n* = 30, 76.9%) considered EM to be very important and a priority in ICU care. However, there was a conservative approach to the level of activity allowed for patients, especially those on ventilatory support. Of the 94 responses to multiple option questions, participants reported they would initiate mobilisation as soon as the patient’s cardio-respiratory status stabilised (*n* = 31, 33%) or as soon as possible after ICU admission (*n* = 15, 16%).

### Perceived contraindications/precautions to early mobilisation

Participants were asked to indicate the highest patient activity level they would allow for a previously ambulatory patient, currently physiologically stable, on mechanical ventilation, on no inotropes and minimally sedated in various scenarios. Online Appendix 2 Table 1-A2 indicates all the responses with only the main findings summarised in the text.

Almost half of the participants would restrict head trauma patients with raised intracranial pressure (*n* = 17, 45.9%) to bedrest, whereas those without raised intracranial pressure were allowed passive range of motion exercises (*n* = 12, 32.4%). Many scenarios were thought to be best managed with bed rest, including patients with cervical (*n* = 12, 30.8%) or thoracolumbar (*n* = 13, 35.1%) injuries, on an intra-aortic balloon pump (*n* = 16, 44.4%), and within 24 h of a treated myocardial infarction (with persistently elevated cardiac enzymes) (*n* = 20, 54). Some felt (*n* = 9, 23.7%) that walking should be allowed in patients with decreasing cardiac enzymes. A third (*n* = 13, 34.2%) would allow transfer to chair within 24 h after uncomplicated cardiac surgery. Some would allow passive range of motion in patients with deep vein thrombosis while on anti-coagulation therapy (*n* = 11, 29.7%), while a similar percentage (*n* = 10, 27%) felt this type of patient should be walking. Most participants (*n* = 24, 61.5%) felt walking should be allowed in obese and frail patients (*n* = 11, 30.6%).

Some scenarios specified lines and attachments frequently used in ICU care, which yielded a wide range of recommended mobility levels. While on mechanical ventilation via endotracheal tube, the highest recommended level of mobility recommended by 11 (28.2%) respondents was active range of motion exercises, with the same percentage (28.2%) indicating that that they would allow transfers to a chair if ventilated via tracheostomy (*n* = 11, 28.2%). Most participants would allow walking with a chest tube (*n* = 14, 36.8%), urinary catheter (*n* = 24, 63.4%), subclavian dialysis line (*n* = 20, 54.1%) and a femoral dialysis line (*n* = 16, 42.1%) during non-dialysis periods. While others would restrict those with femoral central venous catheters (*n* = 12, 31.6%) and radial arterial catheters (*n* = 11, 29.7) to passive range of motion exercises only. Most participants expressed uncertainty towards mobilisation modalities appropriate while receiving extra corporeal membrane oxygenation (*n* = 21, 60%) and high frequency oscillation ventilation (*n* = 18, 50%).

Participants further indicated their opinion on maximum mobility levels based on scenarios including various cardiovascular, respiratory and neurological statuses. These results are reflected in Online Appendix 2, Table 2-A2. Main findings included that more than half of the participants felt that patients receiving high levels of cardiovascular support should be restricted to bedrest (*n* = 19, 51.3%), some allowing higher activity levels as cardiovascular support decreases while others conservatively recommended that patients on no vasopressors or inotropic support should engage in passive range of motion exercise only (*n* = 7, 18.9%). Most would limit patient activity levels to passive range of motion regardless of the level of respiratory support.

### Perceived barriers to early mobilisation

The second part of the survey investigated the perceived barriers towards EM. The main institutional barriers identified were the requirement for a doctor’s referral (*n* = 22, 64.7%), the lack of written guidelines or protocols (*n* = 16, 47.1%) and routine bedrest orders on admission (*n* = 13, 38.2%). Patient-level barriers included medical instability (*n* = 24, 72.7%), excessive sedation (*n* = 18, 54.5%), and endotracheal intubation (*n* = 14, 42.4%) (Table 2).

**TABLE 2:** Institutional and patient-level barriers.

Variable	Total number of responses		Nurses		Doctors		Physiotherapists	
	*n*	%	*n*	%	*n*	%	*n*	%
**Institutional level barriers**	**34**	**100.0**	**17**	**100.0**	**5**	**100.0**	**12**	**100.0**
Routine bedrest orders on admission	13	38.2	5	29.4	1	20.0	7	58.3
Physician orders required prior to mobilisation	22	64.7	10	58.8	1	20.0	11	91.7
Insufficient equipment for EM	9	26.5	5	29.4	0	0.0	4	33.3
No written guidelines or protocols for mobilisation	16	47.1	8	47.1	1	20.0	7	58.3
Not enough physical space	1	2.9	1	5.9	0	0.0	0	0.0
No clinician champion to promote EM in ICU	13	38.2	5	29.4	0	0.0	8	66.7
Perceived an expensive intervention by administrators or unit leaders	1	2.9	0	0.0	1	20.0	0	0.0
No institutional barriers	7	20.6	3	17.6	3	60.0	1	8.3
Other institutional barriers (different physicians seeing patients with poor understanding of need for EM)	1	2.6	0	0.0	0	0.0	1	8.3
**Patient-level barrier**	**33**	**100.0**	**17**	**100.0**	**4**	**100.0**	**12**	**100.0**
Medical instability	24	72.7	13	76.5	3	75.0	8	66.7
Endotracheal intubation	14	42.4	6	35.3	3	75.0	5	41.7
Physical restraints	7	21.2	3	17.6	0	0.0	4	33.3
Risk of dislodgement of devices or lines	13	39.4	7	41.2	2	50.0	4	33.3
Cognitive impairment and/or cognitive age	6	18.2	0	0.0	2	50.0	4	33.3
Excessive sedation	18	54.5	6	35.3	2	50.0	10	83.3
Delirium	5	15.2	1	5.9	2	50.0	2	16.7
Inadequate analgesia	9	27.3	4	23.5	2	50.0	3	25.0
Obesity	2	6.1	0	0.0	1	25.0	1	8.3
Frailty	7	21.2	2	11.8	1	25.0	4	33.3
Inadequate nutritional status	1	3.0	1	5.9	0	0.0	0	0.0
No patient barriers	5	15.2	3	17.6	1	25.0	1	8.3

EM, early mobilisation; ICU, intensive care unit.

All the listed provider level barriers were perceived as barriers by 60% and more of the participants (Table 3). The main three barriers were that EM in the ICU is generally supported but is not perceived as important by some individuals (78.3%), slow to recognise when patients should begin mobilising (77.8%) and safety concerns regarding EM (77.8%).

**TABLE 3:** Provider level barriers.

Provider level barriers	Total/total number of responses	Total %	Nurses (*n* = 21)		Doctors (*n* = 5)		Physiotherapists (*n* = 13)	
			*n*	%	*n*	%	*n*	%
Limited staffing to routinely mobilise patients	15/25	60.0	9	42.9	2	40.0	4	30.8
EM in the ICU is generally supported but not perceived as a priority	16/23	69.6	5	23.8	1	20.0	10	76.9
EM in the ICU is generally perceived as important but is not supported by some individuals	18/23	78.3	7	33.3	1	20.0	10	76.9
A lack of communication among clinician groups to facilitate EM during bedside rounds	17/24	70.8	6	28.6	2	40.0	9	69.2
A lack of communication about rehabilitation during handover at shift change	19/26	73.1	11	52.4	0	8.0	61.5	-
A lack of co-ordination among providers to provide EM	16/27	59.3	7	33.3	1	20.0	8	61.5
Slow to recognise when patients should begin EM	21/27	77.8	9	42.9	2	40.0	10	76.9
A lack of decision-making authority to initiate EM	20/26	76.9	10	47.6	1	20.0	9	69.2
Conflicting perceptions of suitability of EM for patients	20/26	76.9	8	38.1	2	40.0	10	76.9
Safety concerns about EM	21/27	77.8	10	47.6	2	40.0	9	69.2
Inadequate training to facilitate EM	18/25	72.0	9	42.9	1	20.0	8	61.5

EM, early mobilisation; ICU, intensive care unit.

## Discussion

Our study aimed to explore the knowledge, attitudes and practices of nurses, doctors and physiotherapists regarding EM of critically ill patients in Windhoek ICUs. The primary findings indicate knowledge gaps among healthcare professionals regarding EM, conservative mobility practices and numerous perceived barriers to EM implementation. Secondary findings highlight the lack of standardised mobility protocols and the need for targeted education and training programmes to improve EM practices.

### Perspectives on early mobilisation practices

The survey revealed substantial knowledge gaps among healthcare professionals regarding familiarity with EM literature, the incidence of ICUAW and the benefits of EM, which could affect their motivation to prioritise EM. Limited EM knowledge aligns with previous studies that have identified a lack of awareness and familiarity with EM guidelines among ICU staff (Akhtar & Deshmukh 2021; Dagnachew et al. 2023; Dubb et al. 2016). The underestimation of ICUAW incidence and the lack of familiarity with EM literature suggest that healthcare professionals may not fully appreciate the importance of EM, which could hinder its implementation. These findings are consistent with previous studies that have highlighted the need for targeted education and training programmes to improve knowledge and confidence in mobilising critically ill patients (Babazadeh et al. 2021; Hodgson et al. 2021).

A considerable proportion of physiotherapists and nearly half of the nurses reported feeling only somewhat trained to mobilise mechanically ventilated patients, which is consistent with findings from other studies emphasising the need for better training and interprofessional collaboration to facilitate EM (Akhtar & Deshmukh 2021; Dubb et al. 2016). Overall, attitudes towards EM were positive, with most considering it essential for ICU care. This aligns with other studies reporting favourable views on EM (Akhtar & Deshmukh 2021; Babazadeh et al. 2021; Dagnachew et al. 2023; Wang et al. 2020), underscoring its importance for practical implementation. Conservative practices remain common, particularly for patients on ventilatory support, highlighting a broader cultural issue in ICUs where safety concerns frequently overshadow the perceived advantages of EM (Bennion et al. 2024). This aligns with earlier findings suggesting that mechanical ventilation often restricts EM efforts (Capell et al. 2019; Dubb et al. 2016; Fontela, Forgiarini & Friedman 2018; Tadyanemhandu et al. 2021b). This cautious approach may stem from safety concerns and insufficient training, as found in other studies (Babazadeh et al. 2021; Bennion et al. 2024).

Furthermore, our study revealed significant barriers at the patient, provider and institutional levels, which hinder the consistent implementation of EM practices. These three barrier levels are discussed individually in relation to our study findings and previously published literature.

### Patient barriers

Patient-related perceived barriers were prominent in our study, with medical instability, excessive sedation and endotracheal intubation being the most frequently cited obstacles. These findings align with previous literature, which identifies deep sedation and haemodynamic instability as major impediments to EM (Akhtar & Deshmukh 2021; Babazadeh et al. 2021; Lin et al. 2020; Sakuramoto et al. 2023; Tadyanemhandu et al. 2021b; Watanabe et al. 2023).

Patient-related barriers can be further divided into modifiable and non-modifiable barriers (Parry, Nydahl & Needham 2018). Modifiable patient-related barriers to EM are those factors that can be altered or managed through clinical interventions and changes in practice. These include excessive sedation, delirium and inadequate pain management, which can be addressed through optimised sedation protocols, delirium prevention strategies and effective pain control measures recommended as part of the Assess, prevent, and manage pain (A), Both sponta-neous awakening trials (SAT) and spontaneous breathing trials (SBT) (B), Choice of analgesia and sedation (C), Delir-ium: Assess, prevent and manage (D), Early mobility and exercise (E), and Family engagement and empowerment (F) (ABCDEF) ICU liberation bundle (Parry et al. 2017; Sosnowski et al. 2023). On the other hand, non-modifiable patient-related barriers are intrinsic factors that cannot be changed through medical intervention. These include the patient’s underlying medical conditions such as severe haemodynamic instability, profound respiratory failure and certain neurological impairments (Costa et al. 2017). While these non-modifiable barriers present significant challenges, understanding and differentiating them from modifiable barriers allows healthcare providers to focus on what can be controlled and improved, thereby enhancing the overall mobilisation efforts and outcomes for critically ill patients.

### Provider barriers

Provider-related barriers were rated highest among all barrier categories and included factors such as limited support for EM among some ICU staff, delays in recognising when to start EM, safety concerns, the lack of authority to initiate EM and differing views on patient suitability for EM. The prominence of provider-related barriers in our study is consistent with prior literature highlighting the critical role healthcare professionals play in facilitating or impeding EM implementation. A systematic review identified several provider-related barriers, including low confidence levels among physiotherapists, inadequate ICU-specific training and attitudes that do not prioritise EM (Dubb et al. 2016). The limited support for EM among some ICU staff observed in our study aligns with published findings, emphasising that ICU culture and staff attitudes not prioritising early mobility can significantly impede implementation efforts (Albarrati et al. 2024b; Dubb et al. 2016; Tadyanemhandu et al. 2021a).

Delays in recognising when to start EM and differing views on patient suitability for mobilisation were also prominent barriers in our findings. These issues have been previously documented in the literature, with studies highlighting the need for clear protocols and guidelines to assist healthcare providers in identifying appropriate candidates for EM (Dirkes & Kozlowski 2019). The development and implementation of standardised mobility protocols have been suggested as effective strategies to address these barriers and promote consistent EM practices across ICU teams (Gatty et al. 2022; Singam 2024).

Safety concerns emerged as another significant provider-related barrier in our study, which aligns with previous research (Babazadeh et al. 2021; Bennion et al. 2024), despite growing evidence supporting the safety and feasibility of EM in critically ill patients, provided that appropriate protocols are followed (Nydahl et al. 2017; Zhang et al. 2019). Enhancing provider education and establishing clear protocols can help mitigate these barriers and promote a more proactive approach to EM (Babazadeh et al. 2021; Hodgson et al. 2021; Mohamed et al. 2021).

### Institutional barriers

Institutional barriers were also significant, with the requirement for a doctor’s referral, the lack of written guidelines and routine bedrest orders on admission being the most reported obstacles to EM. These findings are in line with previous research that identifies structural barriers such as the absence of protocols, the need for referral and insufficient resources as major impediments to EM (Akhtar & Deshmukh 2021; Babazadeh et al. 2021; Brock et al. 2018; Sakuramoto et al. 2023; Tadyanemhandu et al. 2021b). Implementing institutional changes, such as developing and enforcing EM protocols, increasing staffing, developing regionally appropriate guidelines and designating mobility champions can help overcome these barriers and support the integration of EM into routine ICU care (Tadyanemhandu et al. 2018).

The survey results furthermore indicated that most patients in Windhoek ICUs are not automatically assessed for mobilisation and require a doctor’s referral. Approximately half of the participants reported that their ICUs did not have a mobility protocol or a mobility champion. These findings align with other studies that have identified similar barriers to EM implementation, emphasising the need for multidisciplinary collaboration and the development of clear guidelines to facilitate EM (Costa et al. 2017; Devlin et al. 2018; Dubb et al. 2016; Goddard et al. 2018; Hodgson et al. 2021; Tadyanemhandu et al. 2018). The lack of standardised protocols and reliance on doctor’s referrals can delay the initiation of EM, potentially impacting patient outcomes.

**FIGURE 2:**
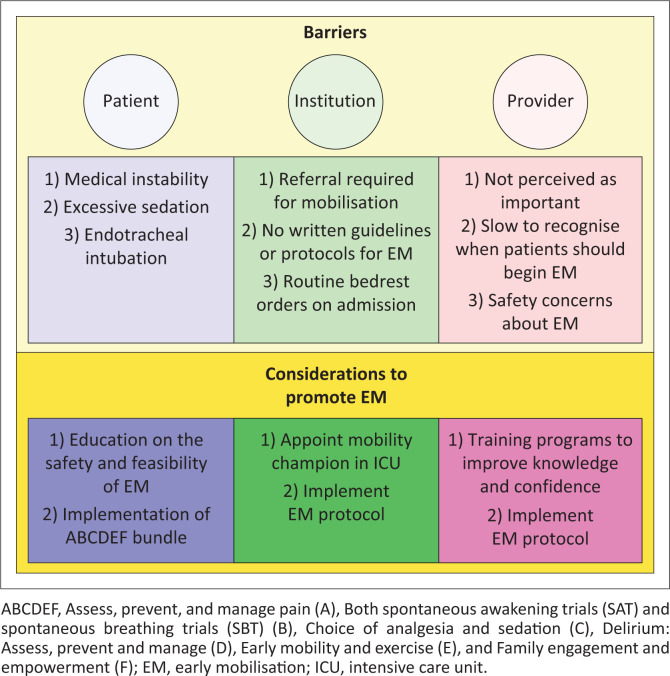
Identified barriers and suggested interventions to promote early mobilisation.

A summary of the main perceived barriers identified in our study and suggested interventions to address these barriers and promote EM are proposed in Figure 2.

### Limitations

Our study has several limitations. The response rate was low and our study therefore underpowered, potentially because of high workload, survey fatigue and limited time availability of healthcare professionals in ICU settings. These factors may limit the generalisability of the findings and may not be truly representative of the ICUs’ EM practices. The population being limited to two private ICUs, the results therefore cannot be generalised to other ICUs in or beyond Windhoek. Additionally, our study relied on self-reported data, which could be subject to response bias as well as a lengthy survey that could have resulted in questionnaire fatigue. Variability in response rates between the online and hard copy surveys might skew results if one method disproportionately represents a particular subgroup. The descriptive nature and cross-sectional design also limits the ability to establish causality between identified barriers and EM practices.

## Conclusion

The findings of our study underscore the importance of addressing knowledge gaps and the multifaceted nature of barriers to EM in Windhoek private ICUs. The identified patient, provider and institutional barriers highlight the need for a comprehensive approach to address these challenges. Clinicians working in ICUs should be aware of the importance of EM and advocate for changes in practice and policy that support its implementation. This includes promoting interprofessional collaboration, enhancing training and education. Implementing standardised mobility protocols and appointing a mobility champion could help overcome institutional barriers and promote a culture of EM. The results suggest that improving knowledge and confidence in EM among healthcare professionals could lead to more proactive mobilisation practices, ultimately improving patient outcomes.

Future research should focus on developing and testing strategies to overcome these barriers and evaluate their impact on patient outcomes. Additionally, exploring the impact of multidisciplinary collaboration and family involvement in EM could provide further insights into optimising mobilisation practices in ICUs. By addressing the identified challenges, healthcare professionals can enhance the quality of care for critically ill patients and optimise their functional recovery.
